# Axial Phosphate Coordination in Co Single Atoms Boosts Electrochemical Oxygen Evolution

**DOI:** 10.1002/advs.202206107

**Published:** 2022-12-09

**Authors:** Yan Liu, Shuangshuang Zhang, Chi Jiao, Huimei Chen, Gang Wang, Wenjie Wu, Zhiwen Zhuo, Junjie Mao

**Affiliations:** ^1^ Key Laboratory of Functional Molecular Solids Ministry of Education College of Chemistry and Materials Science Anhui Normal University Wuhu 241002 P. R. China; ^2^ Institute of Chemistry Chinese Academy of Sciences (CAS) Beijing 100190 P. R. China

**Keywords:** axial coordination, cobalt phthalocyanine, electrocatalysis, oxygen evolution reaction, single atom catalysts

## Abstract

Cobalt single atoms coordinated with planar four nitrogen atoms (Co_1_N_4_) represent an efficient electrocatalyst for oxygen evolution reaction (OER), whereas the large energy barrier of Co—O—H dehydrogenation limits the OER activity. Herein, axial phosphate (PO_4_) coordination is incorporated in Co_1_N_4_ single atoms of cobalt phthalocyanine@carbon nanotubes (P‐CoPc@CNT), so as to boost the intrinsic OER performance through manipulating the reaction pathway. With a relative low mass loading of Co (2.7%), the P‐CoPc@CNT shows remarkable alkaline OER activity with the overpotential of 300 mV and Tafel slope of 41.7 mV dec^−1^, which dramatically outperforms the CoPc@CNT without axial PO_4_ coordination. Based on mechanistic analysis, the axial PO_4_ coordination directly participates in the OER cycle by the transformation of axial ligand. Specially, the Co—O—H dehydrogenation process is replaced by the dehydrogenation of HPO_4_‐Co_1_N_4_ intermediate, which largely decreases the energy barrier and thus benefits the whole OER process.

## Graphical abstract

Axial phosphate (PO_4_) coordination is incorporated in Co_1_N_4_ single atoms of cobalt phthalocyanine@carbon nanotubes (P‐CoPc@CNT), which exhibits remarkable alkaline oxygen evolution reaction (OER) activity. The mechanistic analysis demonstrates that the axial PO_4_ coordination directly participates in the OER cycle, which largely decreases the energy barrier and thus benefits the whole OER process.

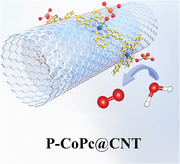

## 1 Introduction

Electrocatalytic oxygen evolution reaction (OER) serves as an important anodic half‐reaction in many energy processes, such as water splitting, CO_2_ reduction, rechargeable metal–air battery, etc.^[^ ^1^, ^2^, ^3^, ^4^, ^5^ ^]^ Generally, the OER suffers from high overpotential and sluggish kinetics, which required the development of highly active electrocatalysts.^[^ ^6^, ^7^, ^8^, ^9^ ^]^ As widely used OER catalysts, various transition‐metal based catalysts have been reported by many researchers.^[^ ^10^, ^11^, ^12^, ^13^ ^]^ Among various OER catalysts, 3d‐transition metal single atoms coordinated with planar four nitrogen atoms (M_1_N_4_) have attracted ever‐increasing attention, due to the high atomic utilization and controllable coordination structure.^[^ ^14^, ^15^, ^16^, ^17^, ^18^ ^]^ In particular, Co_1_N_4_ was highly appealing because of the moderate binding strength for OER intermediates. During the OER process, the Co_1_N_4_ active sites undergo a key procedure of O* formation, which corresponds to the dehydrogenation of the previous OH* intermediate. However, due to the weak acidity of Co—O—H structure, the dissociation of O—H generally requires a large energy barrier, which severely limits the OER activity over Co_1_N_4_ active sites.^[^ ^19^, ^20^, ^21^ ^]^

A promising strategy to promote the OH* transformation is the construction of acid radical as a ligand of Co single atoms, which can be achieved by the introduction of axial coordination. At present, the axial coordination has been developed as an efficient strategy to tailor the performance of M_1_N_4_ single‐atom electrocatalysts.^[^ ^22^, ^23^, ^24^, ^25^, ^26^, ^27^ ^]^ For example, axial Cl coordination on Fe_1_N_4_ single atoms was proven to stabilize the O* and thus promoted the electrocatalytic oxygen reduction reaction (ORR).^[^ ^28^ ^]^ As another example, Co_1_N_5_ active sites with axial N coordination were demonstrated to be an ideal system for the electrochemical reduction of CO_2_ into CO, because of the optimized adsorption of COOH* intermediate.^[^ ^29^ ^]^ In addition, Fe_1_N_4_ single atoms with axial coordination of subsurface O atom was reported to be highly active toward ORR.^[^ ^30^ ^]^ In the system of Co_1_N_4_ for OER, the axial ligand of acid radical was able to provide active center to adsorb the hydrogen‐related intermediates and facilely release the proton due to the ionization effect, and thereby decrease the energy barrier of O* formation. Inspired by the traditional OER catalyst of cobalt phosphate (CoPi), the introduction of axial phosphate (PO_4_) coordination represents a desired strategy to improve the OER performance of Co_1_N_4_ catalysts.

Herein, we implemented the axial PO_4_ coordination on the Co_1_N_4_ sites of cobalt phthalocyanine (CoPc) adsorbed on carbon nanotubes (denoted as P‐CoPc@CNT) for enhanced OER activity. Multiple structural analysis supported the existence of axial PO_4_ coordination on Co_1_N_4_ active centers. Compared with the pristine CoPc@CNT, the P‐CoPc@CNT showed dramatically promoted alkaline OER activity with the overpotential of 300 mV and Tafel slope of 41.7 mV dec^−1^ at a relative low mass loading of Co (2.7%). Mechanistic study revealed that the axial PO_4_ coordination directly participated in the OER cycle, which involved a key intermediate of HPO_4_‐Co_1_N_4_. The weak O—H bond in HPO_4_‐Co_1_N_4_ resulted in a largely deceased energy barrier of dehydrogenation process, which benefited the whole OER process.

## 2 Results and Discussion

**Figure 1:**
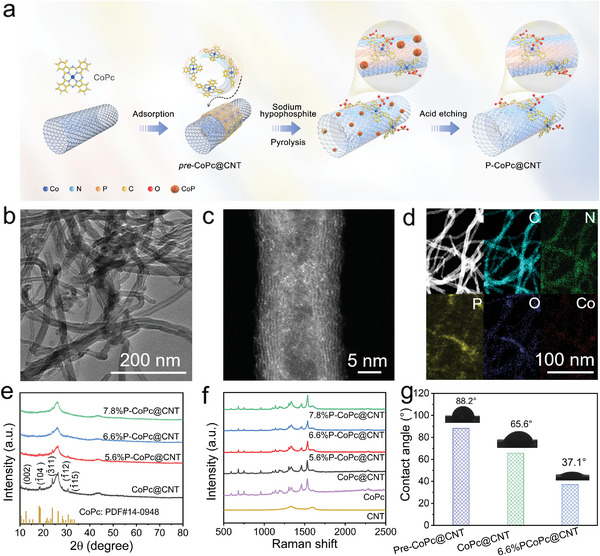
a) Schematic illustration for the synthesis of 6.6%P‐CoPc@CNT, b) TEM image of the 6.6%P‐CoPc@CNT, c) aberration‐corrected (AC)‐HAADF‐STEM image of the 6.6%P‐CoPc@CNT, and d) the corresponding EDS element maps of the 6.6%P‐CoPc@CNT. e) XRD patterns of the CoPc, CoPc@CNT, 5.6%P‐CoPc@CNT, 6.6%P‐CoPc@CNT, and 7.8%P‐CoPc@CNT, f) Raman spectra, and g) Water contact angles of the *Pre*‐CoPc@CNT, CoPc@CNT, and 6.6%P‐CoPc@CNT.

The introduction of axial PO_4_ coordination in CoPc was achieved by a facile pyrolysis procedure (**Figure** **1**a). In a typical synthesis, the CoPc was *pre*‐adsorbed on carbon nanotubes (*pre‐*CoPc@CNT) by *π*–*π* interaction and then mixed with excess sodium hypophosphite. After that, the mixture was then pyrolyzed at 300 °C for 2 h, followed by the removal of phosphide by acid etching. At this temperature, the sodium hypophosphite was decomposed into PO_4_^3−^, whereas the CoPc and CNT were able to retain the pristine structure. Accordingly, the resulting Co single atoms are most likely to distribute on the surface of the CNT, instead of on the bulk phase of the nanotube. When the mass of sodium hypophosphite was 20, 40, and 60 times of *pre‐*CoPc@CNT, the mass percentages of P in the final product were 5.6%, 6.6%, and 7.8%, respectively. As such, the final products were denoted as *wt*%P‐CoPc@CNT. The *pre‐*CoPc@CNT also underwent the similar annealing process to prepare CoPc@CNT for comparison.

The structure and composition of P‐CoPc@CNT was first identified. Regardless of the content of P element, the products remained the nanotube morphology without impurities (Figure 1b and Figure S1, Supporting Information). Taken 6.6%P‐CoPc@CNT as an example, the aberration‐corrected high‐angle annular dark field scanning transmission electron microscopy (HAADF‐STEM) image indicates the isolated distribution of Co species, demonstrating the single‐atom nature (Figure 1c). As shown by the elemental mapping, the signals of C, N, O, P, and Co elements were uniformly distributed in the contour of nanotubes without spacial aggregation (Figure 1d). Determined by energy dispersive spectroscopy (EDS), the content of Co and P were calculated in Table S1 in the Supporting Information which was similar with the inductively coupled plasma optical emission spectrometer (ICP‐OES) data. This result indicated the Co single atoms are rarely distributed on either bulk phase of the CNT, or the bulk phase induced by the close stacking of CNT. In X‐ray diffraction (XRD) patterns, the P‐CoPc@CNT exhibited similar diffraction peaks with CoPc@CNT excepted for the decreased intensity, indicating that the introduction of P element altered the pristine axial stacking of CoPc (Figure 1e). As exhibited by the Raman spectra, all the samples showed the distinct Raman peaks of CoPc molecular (Figure 1f). This result demonstrated that the P element hardly manipulated the bond vibrations of planar CoPc, which was consistent with the regulation of axial coordination by P element. Figure 1g shows the water contact angle measurements of *pre‐*CoPc@CNT, CoPc@CNT, and P‐CoPc@CNT. Compared with the hydrophobic *pre‐*CoPc@CNT, the pyrolysis procedure slightly decreased the water contact angle to 65.6° by surface oxidation. With the decomposition of sodium hypophosphite, the water contact angle dramatically decreased to 37.1°, which further proved the formation of PO_4_^3−^ on the surface. Based on the analysis above, the existence form of P element was most likely to be the axial PO_4_ coordination with Co single atoms.

**Figure 2:**
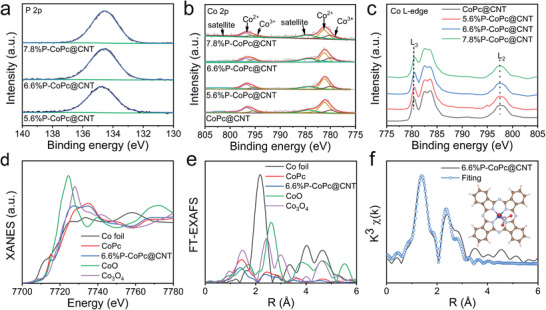
a) P 2p XPS spectra, b) Co 2p XPS spectra, c) XANES Co L‐edge spectra, d) XANES of Co K‐edge, e) Fourier transform (FT) of the Co K‐edge EXAFS spectra of Co foil, CoPc, CoO, Co_3_O_4_, and 6.6%P‐CoPc@CNT, and f) the corresponding EXAFS fitting curve of 6.6%P‐CoPc@CNT in r space. Inset: Schematic model of 6.6%P‐CoPc@CNT (C: brown, N: gray, Co: blue, P: mauve, O: red).

To further validate the axial coordination structure of P‐CoPc@CNT, we carried out the X‐ray photoelectron spectroscopy (XPS) and X‐ray absorption fine structure (XAFS) characterizations. The signals of Co, N, C, O, and P elements were clearly recorded in the XPS survey spectra (Figure S2, Supporting Information). In P 2p XPS spectra, the P‐CoPc@CNT displayed a single P 2p peak at 134.8 eV, which was ascribed to the deeply oxidized P atoms in PO_4_^3−^ (**Figure** **2**a).^[^ ^31^ ^]^ All the P *L*‐edge X‐ray absorption near edge structure (XANES) peaks were located at binding energy (BE) of >135 eV, further verified the oxidation of P (Figure S3, Supporting Information).^[^ ^32^ ^]^ The O 1s XPS of P‐CoPc@CNT revealed the coexistence of P—O (533 eV) and P—O—Co (bridge O atom) (531.5 eV), which indicated the chemical environment of O originated from the axial PO_4_ coordination (Figure S4, Supporting Information). Moreover, the pyrrolic N dominated the N 1s XPS spectra, together with the existence of bridge N and oxidized N, verifying the preservation of CoPc structure (Figure S5, Supporting Information). Additionally, the C 1s and N 1s XANES spectra of CoPc@CNT and P‐CoPc@CNT were very similar, which resulted from the identical CoPc structure (Figure S6, Supporting Information).^[^ ^33^, ^34^ ^]^ By comparison, with the increase of P content, the signal of Co^3+^ was gradually strengthened, which gave rise to the conjunction of PO_4_^3−^ with Co single atoms in CoPc (Figure 2b and Figure S7, Supporting Information). This result was further confirmed by the positive shift of the peak in Co *L3*‐edge XANES spectra (Figure 2c). As a result, the P was existed in the form of axial PO_4_, which bounded with Co single atoms with a Co—O—PO_3_ structure without the alternation of the planar structure of CoPc.

The Co *K*‐edge XAFS experiment was performed to verify the axial PO_4_ coordination with Co_1_N_4_ single atoms. Figure 2d shows the Co *K*‐edge XANES spectra of 6.6%P‐CoPc@CNT and referred species. The 6.6%P‐CoPc@CNT displayed a distinct strong pre‐edge at ≈7715 eV, which originated from the electron transition from Co 1s orbit to 3p*_z_* orbit. Such peak was closely correlated with the symmetry coordination environment of Co single atoms, which was identified as the fingerprint feature of planar Co_1_N_4_. In addition, the pre‐edge signal of 6.6%P‐CoPc@CNT was located at higher binding energy relative to pristine CoPc, demonstrating the slightly oxidation of planar Co_1_N_4_ in CoPc by PO_4_^3−^. Figure 2e shows the Co *K*‐edge extended X‐ray absorption fine structure (EXAFS) spectra of 6.6%P‐CoPc@CNT and referred species. The EXAFS spectrum of 6.6%P‐CoPc@CNT was very similar with that of pristine CoPc except for the shoulder peak accompanied with the Co—N panel. This result was attributed to the axial PO_4_ coordination with the core Co single atom. The EXAFS spectrum of 6.6%P‐CoPc@CNT was further fitted at *R* space, two characterized peaks corresponded to 1.92 and 2.49 Å (phase shift corrected) for bond length (Figure 2f). Such bond lengths were highly consistent with planar Co—N and axial Co—O bonds in the model of CoPc‐PO_3_, which originated from the dehydration of electroneutral hydrogenated PO_4_‐CoPc in the vacuum environment of XAFS experiment. Based on the EXAFS fitting at *k* space and *R* space, the specific coordination number (CN) of 6.6%P‐CoPc@CNT was provided (Figure S8 and Table S2, Supporting Information). The CN for planar Co—N bonds was 3.95, together with the CN of 1.24 for axial Co—O bonds, verifying the local structure of O_2_P—O—Co_1_N_4_ or [O_3_P—O—CoPc]^2−^ in axial PO_4_ coordinated CoPc.

**Figure 3:**
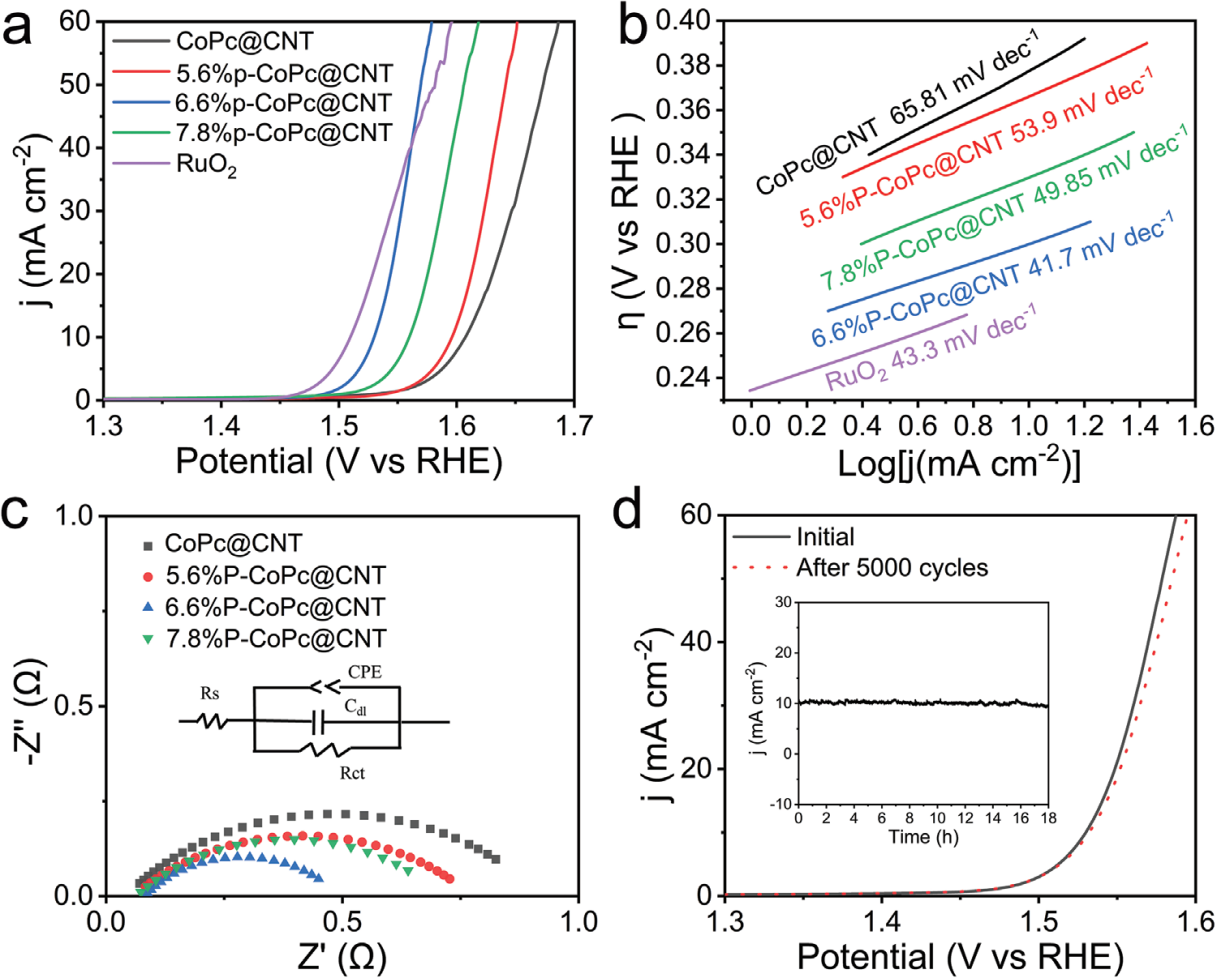
a) Polarization curves, b) Tafel plots, and c) Nyquist plots of pristine CoPc@CNT, 5.6%P‐CoPc@CNT, 6.6%P‐CoPc@CNT, and 7.8%CoPc@CNT. Inset: The fitted equivalent circuit. d) Polarization curves of 6.6%P‐CoPc@CNT before and after 5000 cycles. Inset: *i*–*t* curve of 6.6%P‐CoPc@CNT at the potential of 1.53 V versus RHE.

The tailored axial PO_4_ coordination inspired us to explore the electrochemical OER properties of P‐CoPc@CNT. **Figure** **3**a shows the linear sweep voltammetry (LSV) curves of the CoPc@CNT and P‐CoPc@CNT. The commercial RuO_2_ catalyst was also tested for comparison. With the P content increased from 0% to 6.6%, the OER activity displayed the obvious enhancement. Further increasing the P content to 7.8% led to a decrease in the performance, which was likely attributed to the shielding of surface active sites by excess PO_4_^3−^. Specifically, 6.6%P‐CoPc@CNT possessed a lowest overpotential of 300 mV at 10 mA cm^−2^ among these catalysts, which approached the value of the commercial RuO_2_ catalyst and the recently reported single‐atom catalysts.^[^ ^35^, ^36^, ^37^ ^]^ Considering that the 6.6%P‐CoPc@CNT only contained less than 3% mass content of Co, we performed the calculation of turnover frequency (TOF) values for CoPc@CNT and P‐CoPc@CNT. As shown in Table S3 in the Supporting Information, the TOF of 6.6%P‐CoPc@CNT reached a high value of 0.071 s^−1^, which was an order of magnitude higher than that of CoPc@CNT. As indicated by the Tafel plots of the four catalysts and the commercial RuO_2_, the 6.6%P‐CoPc@CNT showed a lowest Tafel slope of 41.7 mV dec^−1^ (Figure 3b). Such value was much lower than 65.8 mV dec^−1^ of CoPc@CNT, indicating the promoted the OER kinetics by the introduction of P. These results demonstrated that the axial PO_4_ on Co single atom improved the OER performance of CoPc.

The promotion of OER performance by axial PO_4_ was verified by the investigation of charge‐transfer process. Figure 3c shows the Nyquist curves and the equivalent circuit of the CoPc@CNT and P‐CoPc@CNT, where the diameter of the semicircle represented the charge‐transfer resistance (*R*_CT_). The *R*_CT_ of 6.6%P‐CoPc@CNT was 0.3 Ω, which exhibited 57% decrease compared with that of CoPc@CNT (Table S4, Supporting Information). In addition, we explored the double layer capacitance (*C*_dl_) to evaluate the influence of the electrochemical active area on the reaction. Calculated from the cyclic voltammetry (CV) curves at various scan rates, the *C*_dl_ values of CoPc@CNT, 5.6%P‐CoPc@CNT, 6.6%P‐CoPc@CNT, and 7.8%P‐CoPc@CNT were 4.15, 4.55, 4.80, and 3.10 mF cm^−2^, respectively (Figures S9 and S10, Supporting Information). This result indicated the axial PO_4_ tuned the electrochemical active sites of CoPc. Moreover, the *C*_dl_‐normalized OER activity of these four catalysts remained the same trend as the geometrical LSV curves, demonstrating the axial PO_4_ also enhanced the intrinsic activity of Co single atoms (Figure S11, Supporting Information). In the stability evaluation, the 6.6%P‐CoPc@CNT exhibited less than 5% decay during 18 h potentiostatic test and remained a low overpotential of 303 mV at 10 mA cm^−2^ after 5000 cycle CV tests. Furthermore, the transmission electron microscopy (TEM), XRD, ICP, and XPS measurements of 6.6%P‐CoPc@CNT after the potentiostatic test rarely displayed alternation (Figures S12–S14 and Table S5, Supporting Information). These results proved the potential long‐term use of 6.6%P‐CoPc@CNT catalysts.

**Figure 4:**
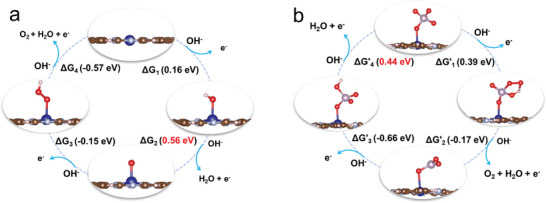
a) The free energy change of the OER process over CoPc at 1.23 V versus RHE and b) the free energy change of the OER process over PO_4_‐CoPc at 1.23 V versus RHE. The blue, mauve, bluish, brown, red, and white spheres represent Co, P, N, C, O, and H, respectively.

Motivated by the facilitated OER kinetics, we further explored the reaction mechanism on the P‐CoPc@CNT by the density function theory calculations. Considering the alkaline electrolyte, the PO_4_‐CoPc model was adopted to simulate the P‐CoPc@CNT. **Figure** **4**a shows the Gibbs free energy change (Δ*G*) of an OER reaction cycle on Co_1_N_4_ active site of a typical CoPc model. The main bottleneck lies on the step of OH*+OH^−^ → O* +H_2_O +e^−^, which required a large Δ*G* value of 0.56 eV. By comparison, the reaction path of OER on axial PO_4_‐Co_1_N_4_ varied due to the direct participation of PO_4_ (Figure 4b). As the first OH^−^ adsorbed on PO_4_‐Co_1_N_4_, the P atom in the intermediate bound with —O_2_, —OH, —O, and —O—Co_1_N_4_. With the introduction of the second OH^−^, O_2_, and H_2_O molecules were able to be desorbed and thus formed PO_3_‐Co_1_N_4_. After that, the PO_3_‐Co_1_N_4_ trapped an additional OH and transformed into PO_4_H‐Co_1_N_4_, followed by the removal of H by OH^−^ in the last step. During the OER cycle, the rate‐limiting step was the dissociation of H in the —OH of PO_4_H‐Co_1_N_4_, which was very similar with the OH* dehydrogenation in the OER cycle on Co_1_N_4_ active site. Notably, the Δ*G* of PO_4_H‐Co_1_N_4_ dehydrogenation was 0.44 V, outperforming the 0.56 eV of OH* dehydrogenation on Co_1_N_4_ (Figure S15 and Table S6, Supporting Information).

To further reveal the facilitated dehydrogenation process, we analyzed the bond energy of the O—H bond in PO_4_H‐Co_1_N_4_ and OH—Co_1_N_4_. The bond energy of O—H in PO_4_H‐Co_1_N_4_ was −1.97 eV, which was less negative than that of OH—Co_1_N_4_ (−2.08 eV). Figure S16 in the Supporting Information shows the barder charge distributions of OH—Co_1_N_4_ and PO_4_H‐Co_1_N_4_. The H atom in PO_4_H‐Co_1_N_4_ and OH—Co_1_N_4_ displayed the positive charges of 0.62 and 0.56 e Bohr^−3^, respectively. Considering the positive charged H atoms tended to be dissociated, the dehydrogenation of PO_4_H‐Co_1_N_4_ was thermodynamically favored relative to that of OH—Co_1_N_4_, thus accounting for the decreased energy barrier in OER process.

## 3 Conclusion

In conclusion, the axial PO_4_ coordination was introduced on Co_1_N_4_ of CoPc for boosted OER performance. The axial PO_4_ coordination on CoPc constructed a local atomic structure of PO_4_‐Co_1_N_4_. With 2.7% mass loading of Co, the 6.6%P‐CoPc@CNT possessed a low overpotential of 300 mV and Tafel slope of 41.7 mV dec^−1^, which dramatically outperformed the CoPc@CNT. During the OER cycle of P‐CoPc@CNT, the PO_4_‐Co_1_N_4_ active sites underwent the transformation of axial ligand, in which the dehydrogenation of HPO_4_‐Co_1_N_4_ intermediate acting as the rate‐limiting step. Due to the weak O—H bond in HPO_4_‐Co_1_N_4_, the energy barrier of dehydrogenation was much lower than traditional OH* dehydrogenation on Co_1_N_4_, which gave rise to the improved OER activity. This work broadened the horizon of tuning reaction path for single‐atom electrocatalysis via engineering the axial coordination.

## 4 Experimental Section

### Chemicals

All chemicals were used without further purification: carbon nanotubes multiwalled (>95%, 3–5 nm inner diameter (ID), 8–15 nm outer diameter (OD), Innochem), sodium hypophosphite (Innochem), cobalt phthalocyanine (92%, J&K), muriatic acid (36%–38%), ethanol (>99.7%), C_3_H_8_O (>99.7%), nafion (5%, Alfa Aescar).

### Synthesis of Pre‐CoPc@CNT

0.1 g of CNT and 0.08 g of CoPc were dissolved in 80 mL ethanol by sonication for 1 h at room temperature. The mixture was stirred at room temperature for 24 h and dried at 60 °C for further use.

### Synthesis of CoPc@CNT

The solid powder pre‐CoPc@CNT was placed in a tubular carbonization furnace and carbonized at 300 °C for 2 h under Ar gas conditions at a heating rate of 2 °C min^−1.^. The obtained sampled is named CoPc@CNT.

### Synthesis of 5.6%P‐CoPc@CNT

The obtained 20 mg pre‐CoPc@CNT and 400 mg sodium hypophosphite were ground together and then calcined at 300 °C for 2 h under Ar gas conditions at a heating rate of 2 °C min^−1^. The mixture was etched by 3 m of HCl for 12 h and then washed by water for three times to obtain the 5.6%P‐CoPc@CNT. The 6.6%P‐CoPc@CNT and 7.8%P‐CoPc@CNT were similarly prepared by following this procedure except for changing the content of sodium hypophosphite into 0.8 and 1.2 g, respectively.

### Characterization

Powder XRD was performed by a Smart Lab X‐ray powder diffractometer with monochromatized Cu *K*_*α*_ radiation *(λ* = 1.5418 Å). TEM was taken on a Hitachi HT7700 working at 100 kV. ICP‐OES was carried out on Aglient 5110. Raman spectra were measured by InVia with a laser wavelength of 532 nm at room temperature. The XPS was conducted on a Thermo Scientific K‐Alpha Quantera microscope. The BE were calibrated by setting the measured BE of C 1s to 284.8 eV. The absorption spectra of Co K‐edge were obtained in the transmission mode using a Si (111) double‐crystal monochromator at the XAFS station of the 1W1B beamline of the Beijing Synchrotron Radiation Facility (BSRF, China) and BL14W1 station in Shanghai Synchrotron Radiation Facility.

### Electrochemical Test

Electrochemical measurements were performed with a CHI760E electrochemical workstation. OER activities were recorded in a three‐electrode system with Ag/AgCl as the reference electrode and a graphite rod as the counter electrode in N_2_‐saturated 1.0 m KOH electrolyte, respectively. To prepare the working electrode, 3 mg of catalyst powder was dispersed in 300 µL of mixed solution (140 µL of water, 150 µL of ethanol, and 10 µL of 5 wt% Nafion solution). After sonication for 20 min, a homogeneous ink was obtained. 10 µL of the ink was coated on a 4 mm glassy carbon electrode. LSV was performed at a scan rate of 5 mV s^−1,^ and the polarization curves were calibrated with 100% iR compensation to eliminate the solution resistance. All potentials measured were converted to a reversible hydrogen electrode (RHE) using the following equation: *E*_versusRHE_ = *E*_versusAg/AgCl_ + 0.197 V + 0.059 pH. Electrochemical impedance spectroscopy was recorded at 1.23 V. The double‐layer capacitances (*C*_dl_) were calculated through cyclic voltammogram (CV) at different scan rates (i.e., 5, 10, 15, 20, and 25 mV s^−1^) in 1.0 m KOH. The potentiostatic test was carried out at the potential of 1.53 V versus RHE for 18 h.

## Conflict of Interest

The authors declare no conflict of interest.

## Supporting information

Supporting Information

## Data Availability

The data that support the findings of this study are available from the corresponding author upon reasonable request.
