# Supplementary material for: Axial Phosphate Coordination in Co Single Atoms Boosts Electrochemical Oxygen Evolution
Source: Adv Sci (Weinh). 2022 Dec 9;10(5):2206107. doi: 10.1002/advs.202206107 (PMC9929106; doi:10.1002/advs.202206107)
Supplement: Supplementary file 1 — Supporting Information [file ADVS-10-2206107-s001.pdf]

ADVANCED SCIENCE
Open Access

Supporting Information

for *Adv. Sci.*, DOI 10.1002/advs.202206107

Axial Phosphate Coordination in Co Single Atoms Boosts Electrochemical Oxygen Evolution

Yan Liu, Shuangshuang Zhang, Chi Jiao, Huimei Chen, Gang Wang, Wenjie Wu, Zhiwen Zhuo and Junjie Mao**

Supporting Information

Axial Phosphate Coordination in Co Single Atoms Boosts Electrochemical Oxygen Evolution

Yan Liu, Shuangshuang Zhang, Chi Jiao, Huimei Chen, Gang Wang, Wenjie Wu, Zhiwen Zhuo, Junjie Mao**
Y. Liu, S. Zhang, C. Jiao, H. Chen, G. Wang, Z. Zhuo, J. Mao
Key Laboratory of Functional Molecular Solids, Ministry of Education, College of Chemistry and Materials Science, Anhui Normal University
Wuhu, 241002, China
E-mail: ly0201@ahnu.edu.cn; maochem@ahnu.edu.cn
W. Wu
Institute of Chemistry, Chinese Academy of Sciences (CAS)
Beijing 100190, China

TOF calculations

TOF=(jA)/(4Fn)

where j is the current density (mA cm^{-2}), A is the geometric area of the electrode (cm^2), 4 is the mole of electrons consumed for evolving one mole of O_2 from water, F is the Faradic constant (96,485 C mol^{-1}), and n is the number of active sites (mol).

Computational details

We performed the DFT calculations by using the Vienna ab initio simulation package (VASP) [1,2]. The Perdew-Burke-Ernzerhof (PBE) [3] functional was used for describing electronic exchange. The projector augmented wave [4,5] method was adopted to describe the interactions between the ion cores and valence electrons. The energy cutoff for the plane-wave basis set was 500 eV. The Brillouin zone was sampled by the Monkhorst−Pack scheme [6] using a 3 × 3× 1 k-point grid. During the geometry optimization and electronic structure calculation, the atomic positions were optimized until the energy and the maximum force were less than 10^{-5} eV/atom and 0.02 eV/Å, respectively. The van der Waals dispersion by employing the DFT-D3 [7] method of Grimme was considered for all the calculation. The Hubbard U (DFT + U) treatment was used on the transition metal, and the U value for Co was set to 3.42, following the literature value.[8] The solvation effect was considered by an implicit solvent model VASPsol with the dielectric constant of 78.4 for water.[9]

The OER process of Co_1N_4 includes four basic steps:

$* + OH^- \rightarrow OH^* + e^-$ (1)

$OH^* + OH^- \rightarrow O^* + H_2O + e^-$ (2)

$O^* + OH^- \rightarrow OOH^* + e^-$ (3)

$OOH^* + OH^- \rightarrow * + O_2 + H_2O + e^-$ (4)

where * represents an active site on the bare catalysts surface, and OH*, O*, OOH* represent three different catalytic intermediates.

The PO_4-Co_1N_4 OER process includes four basic steps:

$PO_4\text{-}Co_1N_4 + OH^- \rightarrow PO_5H\text{-}Co_1N_4 + e^-$ (1')

$PO_5H\text{-}Co_1N_4 + OH^- \rightarrow PO_3\text{-}Co_1N_4 + O_2 + H_2O + e^-$ (2')

$PO_3\text{-}Co_1N_4 + OH^- \rightarrow PO_4H\text{-}Co_1N_4 + e^-$ (3')

$PO_4H\text{-}Co_1N_4 + OH^- \rightarrow PO_4\text{-}Co_1N_4 + H_2O + e^-$ (4')

Based on these reaction proceeds, the Gibbs free energy changes are calculated as follows:

$\Delta G = \Delta E + \Delta ZPE - T\Delta S$

where ΔE is the total energy difference between reactants and products of reactions, ΔZPE is the zero-point energy correction, ΔS is the vibrational entropy change at finite temperature T. The overpotential η can be evaluated from the Gibbs free energy differences of each step as: $\eta = \max[\Delta G_1, \Delta G_2, \Delta G_3, \Delta G_4] /e - 1.23$ or $\max[\Delta G_1', \Delta G_2', \Delta G_3', \Delta G_4'] /e - 1.23$.

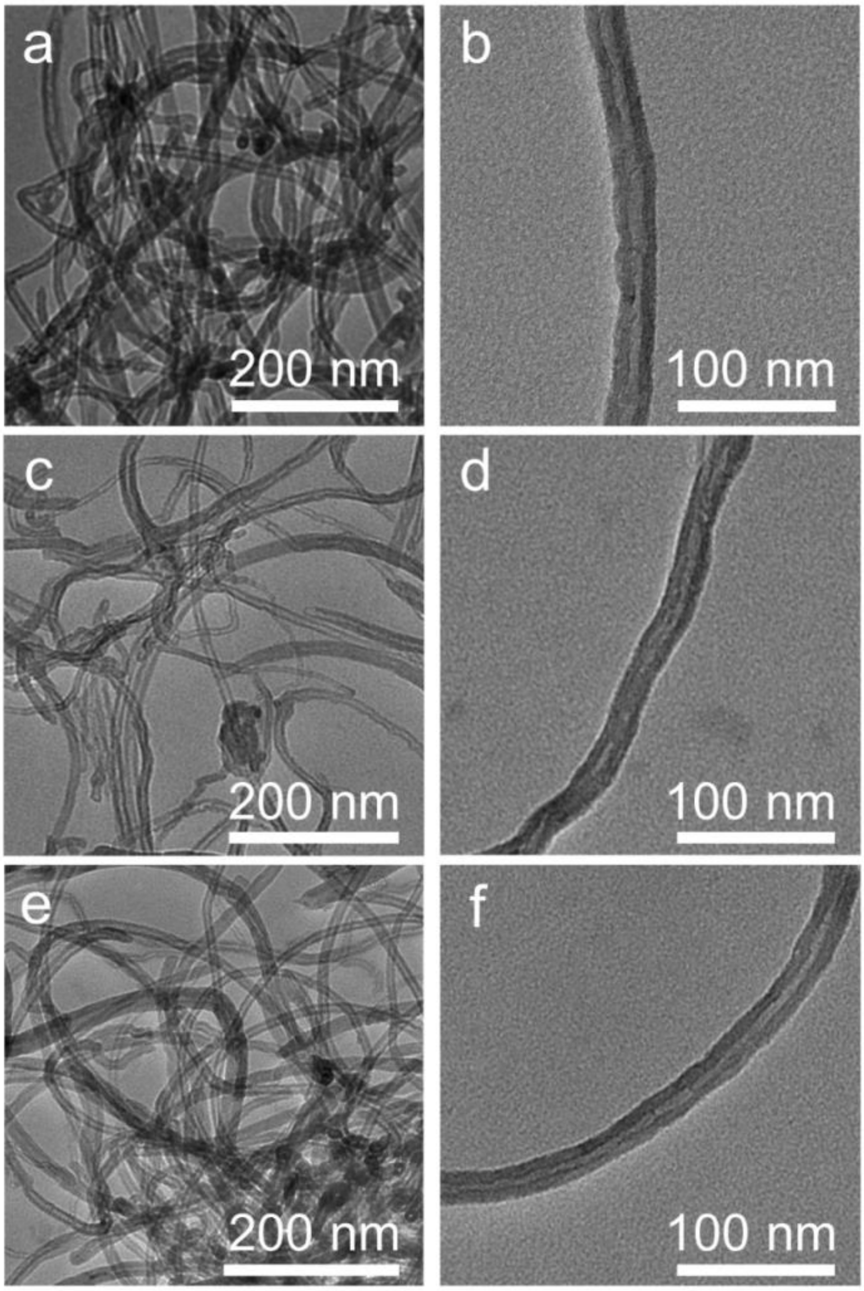

Figure S1. The TEM characterizations of (a,b) CoPc@CNT, (c,d) 5.6%P-CoPc@CNT, (e,f) 7.8%P-CoPc@CNT.

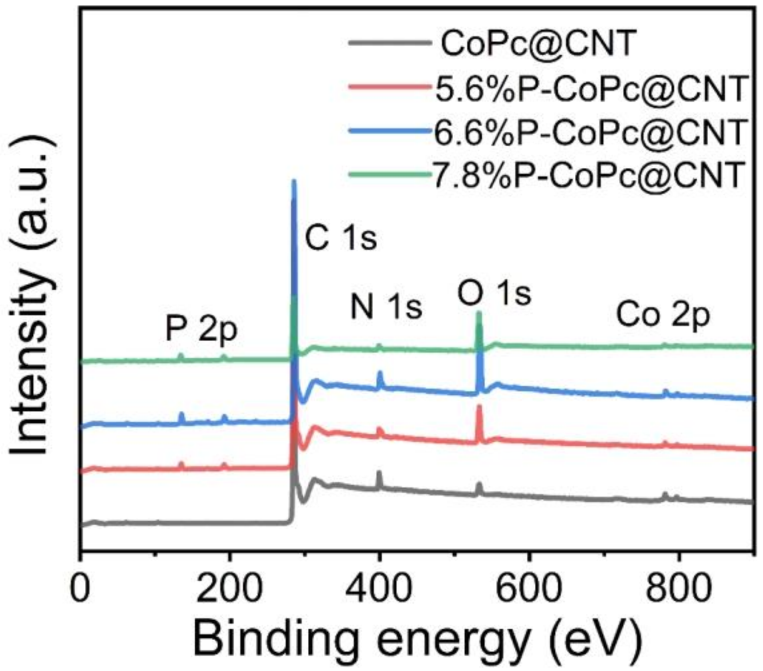

Figure S2. XPS survey spectra of CoPc@CNT and P-CoPc@CNT.

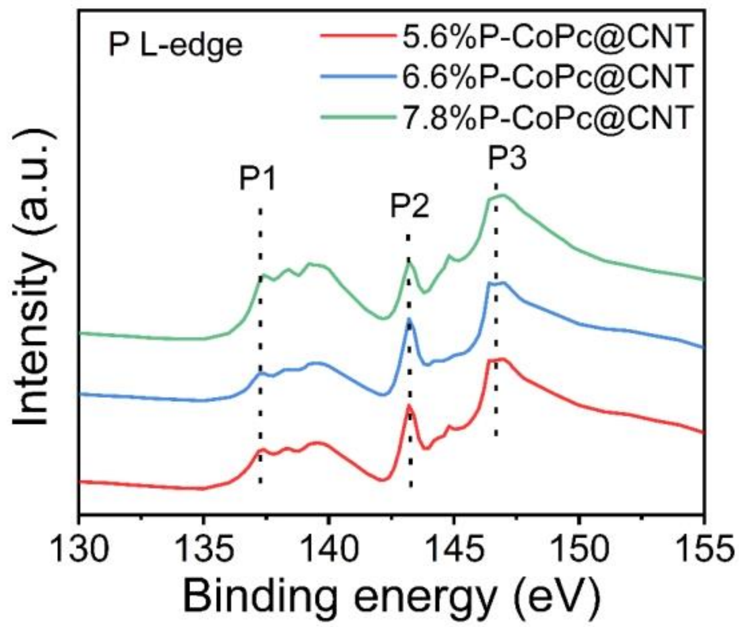

Figure S3. P L-edge XANES spectra of P-CoPc@CNT.

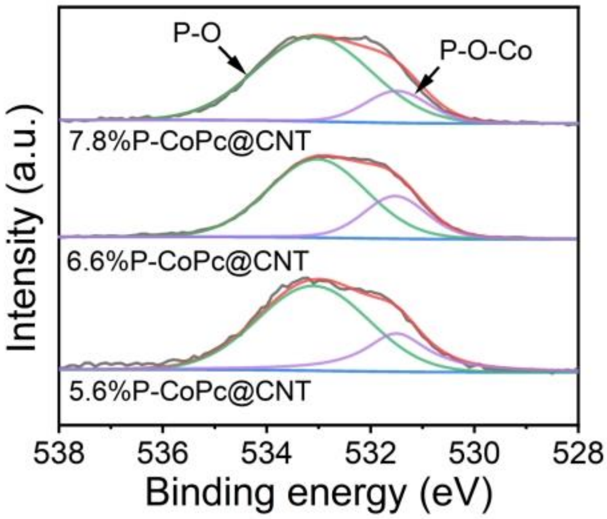

Figure S4. O 1s XPS spectra of P-CoPc@CNT.

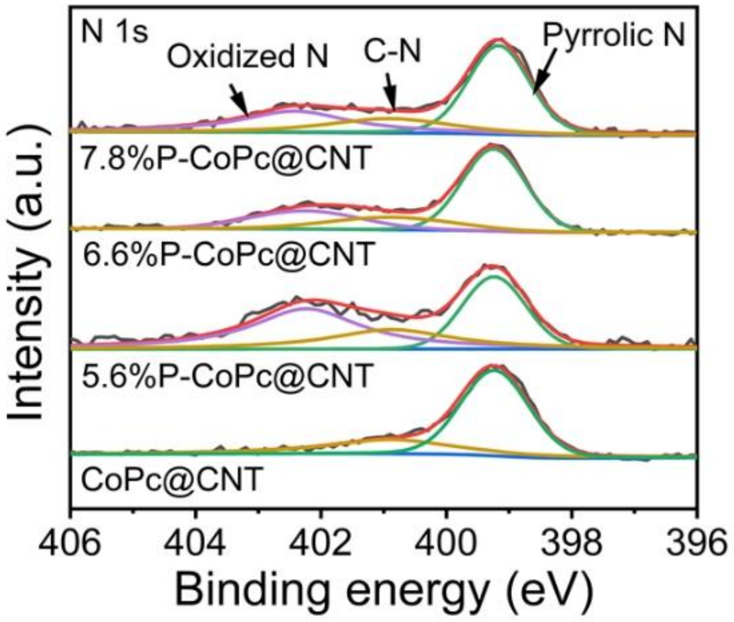

Figure S5. N 1*s* XPS spectra of CoPc@CNT and P-CoPc@CNT.

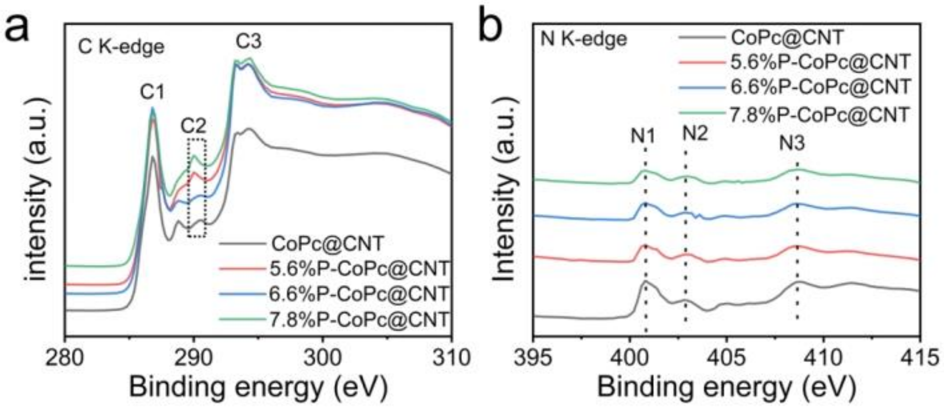

Figure S6. The (a) C K-edge XANES and (b) N K-edge XANES of CoPc@CNT and P-CoPc@CNT.

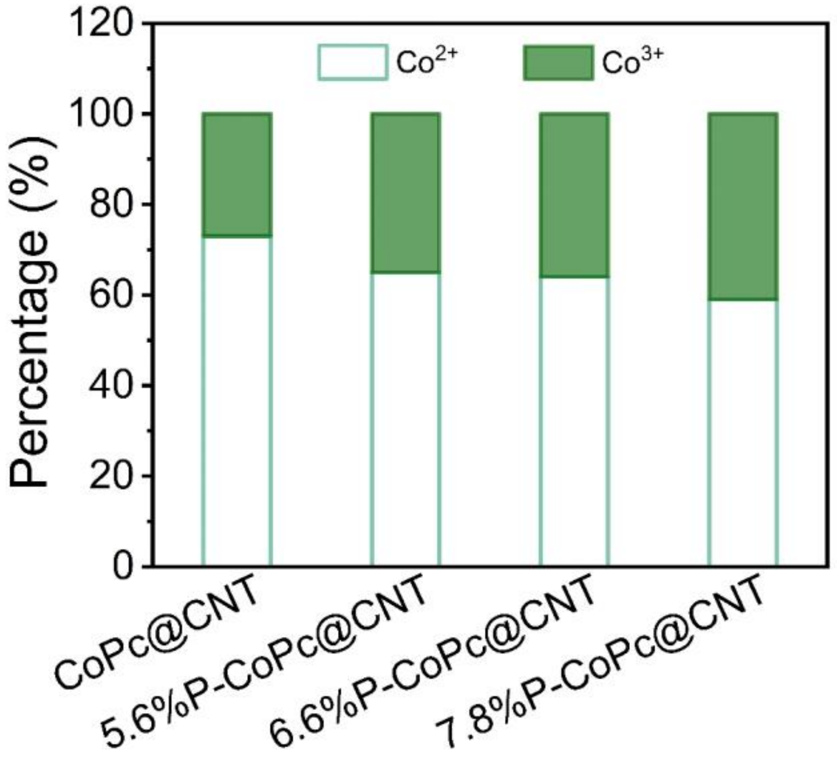

Figure S7. Percentage of Co^{2+} and Co^{3+} in CoPc@CNT and P-CoPc@CNT, which calculated by Co 3*p* XPS spectra.

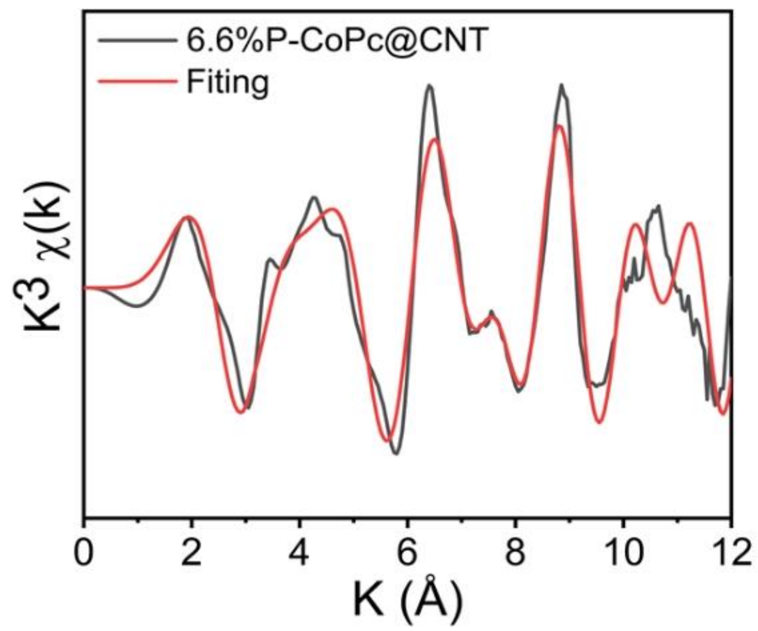

Figure S8. The corresponding EXAFS fitting curve of 6.6%P-CoPc@CNT in *k* space.

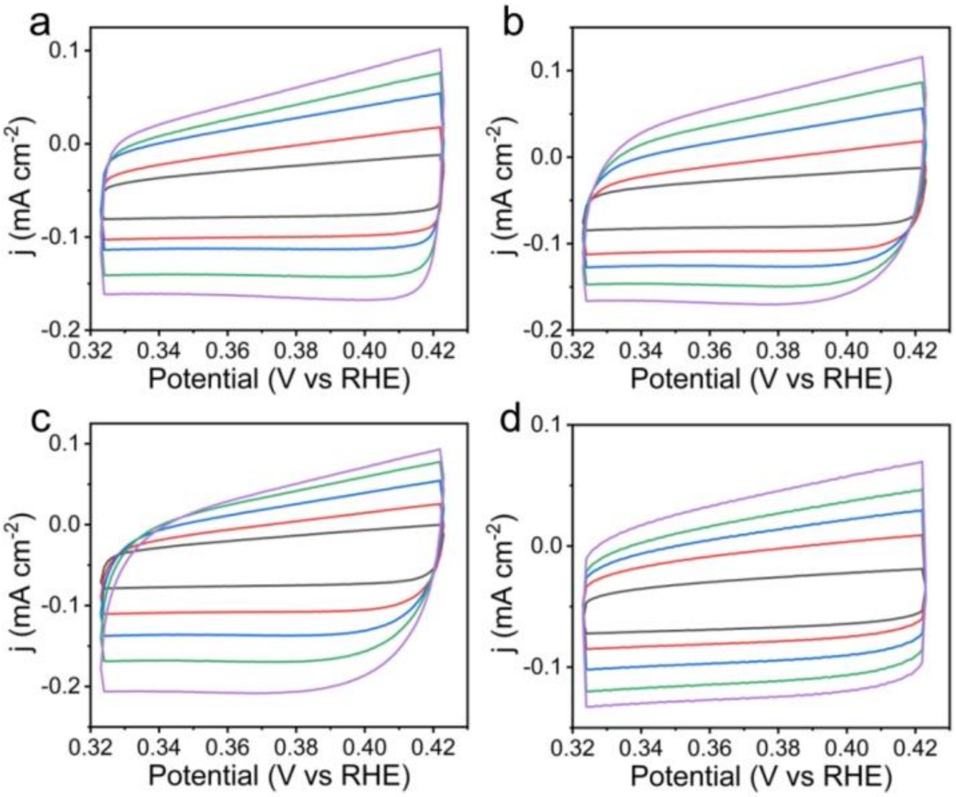

Figure S9. Cyclic voltamogram curves of (a) pristine CoPc@CNT, (b) 5.6%CoPc@CNT, (c) 6.6%CoPc@CNT, and (d) 7.8%CoPc@CNT in the double layer capacitive region at scan rates of 5-25 mV s^{-1}.

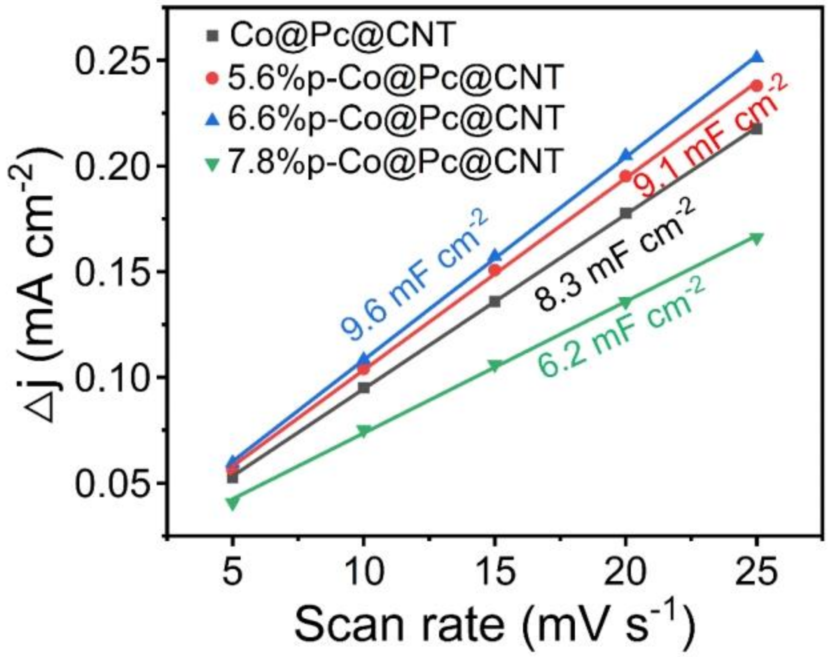

Figure S10. The range of non-Faradaic processes for measuring value of C_{dl} (the slope equals twice C_{dl}).

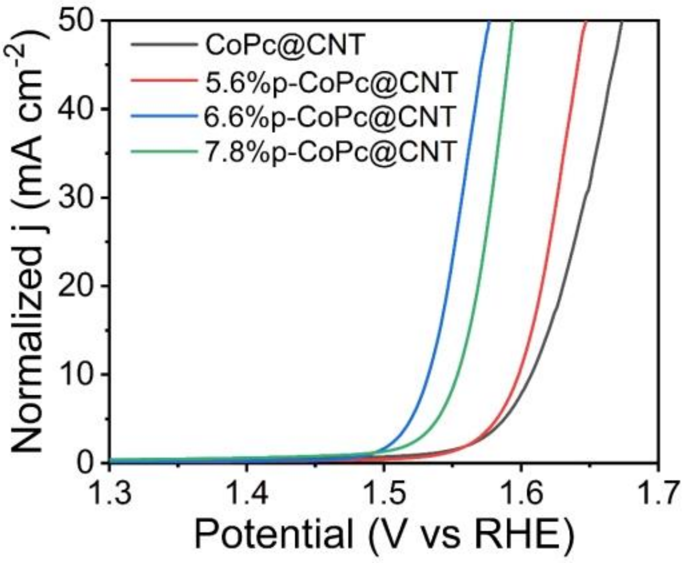

Figure S11. Current densities (*j*) normalized by the $C_{dl,\ P\text{-}CoPc@CNT}/C_{dl,\ CoPc@CNT}$ values over CoPc@CNT and P-CoPc@CNT.

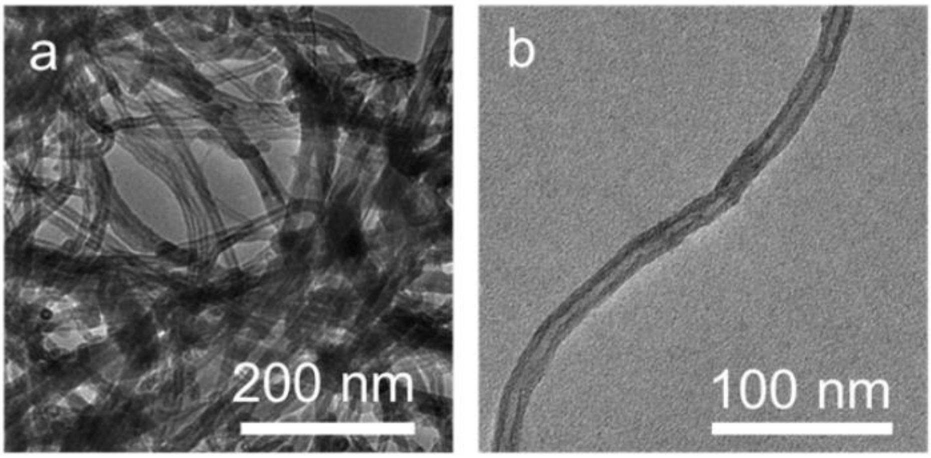

Figure S12. TEM images of 6.6%P-CoPc@CNT after the potentiostatic test.

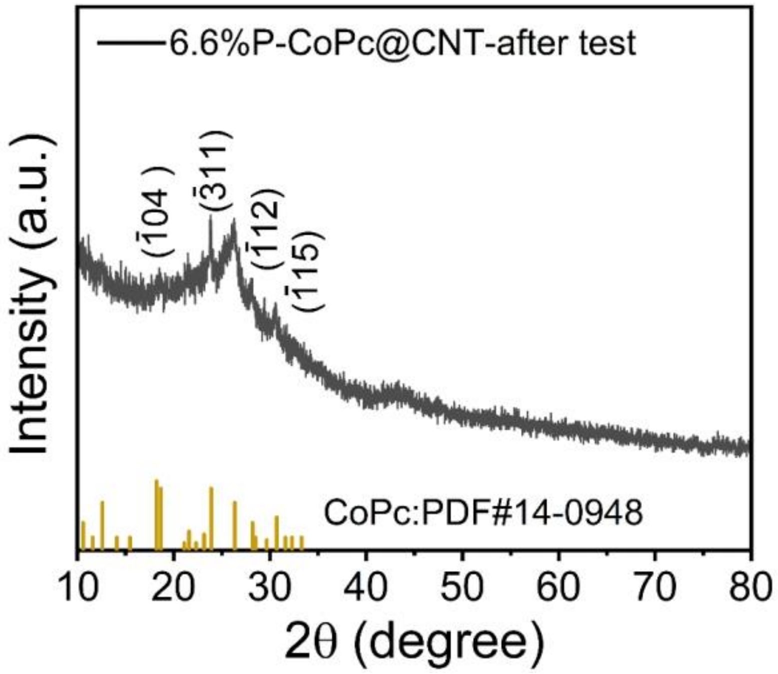

Figure S13. XRD pattern of 6.6%P-CoPc@CNT after the potentiostatic test.

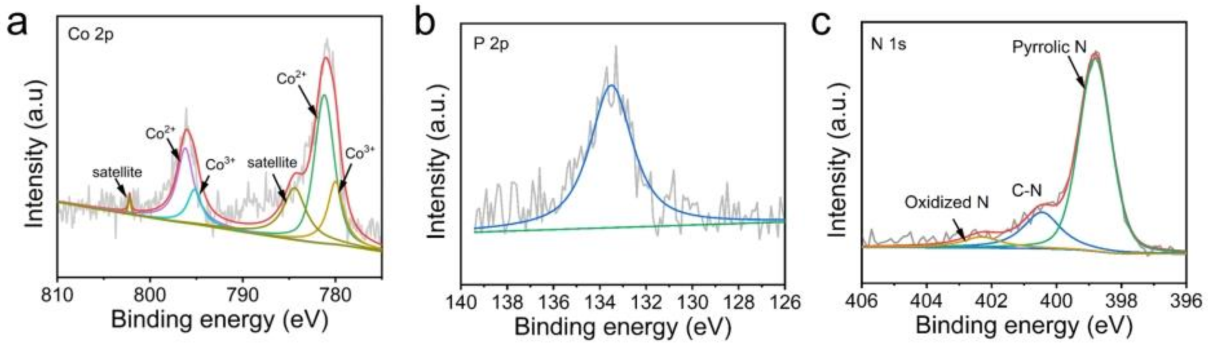

Figure S14. The XPS spectrum of 6.6%P-CoPc@CNT after the potentiostatic test. (a) Co 2*p*, (b) P 2*p*, and (c) N 1s.

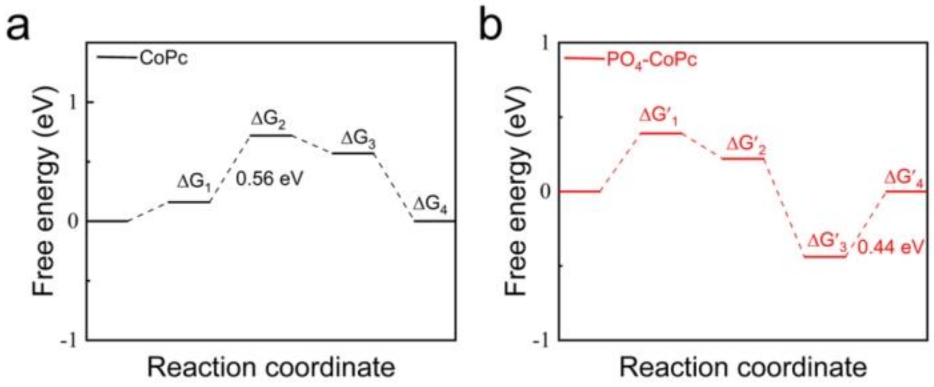

Figure S15. The diagram of Gibbs free energy changes for the four elementary OER step over CoPc and PO_4-CoPc models.

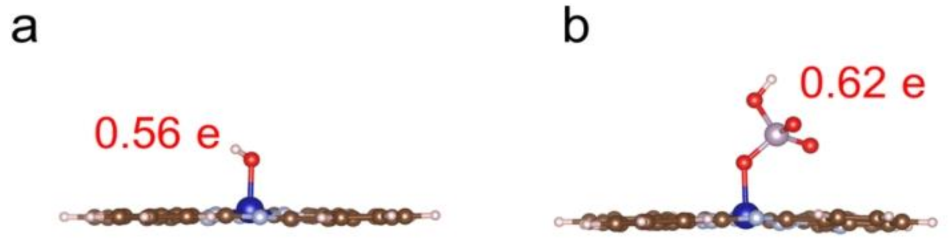

Figure S16. The barder charge distributions of (a) OH-Co_1N_4 and (b) PO_4H-Co_1N_4.

Table S1. The mass content of Co and P in electrocatalysts determined by EDS and ICP-MS.

Sample	Co (%) EDS	P (%) EDS	Co (%) ICP	P (%) ICP
CoPc@CNT	4.03	-	3.5	-
5.6%P-CoPc@CNT	3.01	5.35	2.9	5.6
6.6%P-CoPc@CNT	2.26	6.93	2.7	6.6
7.8%P-CoPc@CNT	2.15	7.64	2.4	7.8

Table S2. Structural parameters extracted from the Co K-edge EXAFS fitting. (S_0^2=0.71)

Sample	Scattering pair	CN	R(Å)	σ^2(Å^2)	ΔE_0(eV)	R factor
Co foil	Co-Co	12*	2.49	0.006	6.513	0.002
	Co-N	3.95	1.92	0.005		
CoPc/P-CNT	Co-O	1.24	2.16	0.006	-1.18	0.029
	Co-C	4.16	2.94	0.032		

S_0^2 is the amplitude reduction factor; CN is the coordination number; R is interatomic distance (the bond length between central atoms and surrounding coordination atoms); σ^2 is Debye-Waller factor (a measure of thermal and static disorder in absorber-scatterer distances); ΔE_0 is edge-energy shift (the difference between the zero kinetic energy value of the sample and that of the theoretical model). R factor is used to value the goodness of the fitting.

* This value was fixed during EXAFS fitting, based on the known structure.

Error bounds that characterize the structural parameters obtained by EXAFS spectroscopy were estimated as N ± 5%; R ± 1%; σ^2 ± 20%; ΔE_0 ± 20%.

CoPc/CNT (FT range: 2.0-10.30 Å^{-1}; fitting range: 1.14-3.3 Å)

Co foil (FT range: 3.0-12.0 Å^{-1}; fitting range: 1.0-3.0 Å)

Table S3. Comparison of the TOF values at the overpotential of 300 mV over the catalysts.

**Sample**	**TOF** (s^{-1})
CoPc@CNT	5.6×10^{-3}
5.6%P-CoPc@CNT	5.3×10^{-3}
6.6%P-CoPc@CNT	7.1×10^{-2}
7.8%P-CoPc@CNT	2.0×10^{-2}

Table S4. The calculated Rs and Rct values over the catalysts, which were fitted by the equivalent circuit in Figure 3c.

**Sample**	**R_s (Ω,)**	**R_{ct} (Ω)**
CoPc@CNT	1.2	0.7
5.6%P-CoPc@CNT	2.9	0.6
6.6%P-CoPc@CNT	2.7	0.3
7.8%P-CoPc@CNT	2.8	0.6

Table S5. The mass content of Co and P in pristine 6.6%P-CoPc@CNT and the catalyst after potentialstatic test by ICP-OES.

Sample	Co (%)	P (%)
6.6%P-CoPc@CNT	2.7	6.6
6.6%P-CoPc@CNT-after test	2.5	6.5

Table S6. Calculated reaction free energy for OER intermediates

Sample	ΔG_1	ΔG_2	ΔG_3	ΔG_4	η/V
CoPc	0.16	0.56	-0.15	-0.57	0.56
PO_4-CoPc	0.39	-0.17	-0.66	0.44	0.44

References

[1] G. Kresse, J. Furthmiiller, *Comput. Mater. Sci*. **1996**, *6*, 15.

[2] G. Kresse, J. Furthmiiller. *Matter Mater. Phys.***1996** ,*54*, 11169.

[3] J. Perdew, K. Burke, M. Ernzerhof, *Phys. Rev. Lett.* **1996**, *77*, 3865.

[4] P. Blöchl, *Phys. Rev. B.* **1994**, *50*, 17953.

[5] G. Kresse., D. Joubert, *Matter Mater. Phys.* **1999**, *59*, 1758.

[6] H. Monkhorst, D. Pack, *Phys. Rev. B.* **1976**, *13*, 5188.

[7] S. Grimme, J. Antony, S. Ehrlich, H. Krieg, *J. Chem. Phys*. **2010**, *132*, 154104.

[8] S. Dudarev, G. Botton, S. Savrasov, C. Humphreys, A. Sutton, *Phys. Rev. Lett*. **1998**, *57*, 1505.

[9] K. Mathew, R. Sundararaman, K. Letchworth-Weaver, T. Arias, R. Hennig, *J. Chem. Phys*. **2014**, *140*, 084106.
